# Cytauxzoonosis in North America

**DOI:** 10.3390/pathogens10091170

**Published:** 2021-09-10

**Authors:** Mason V. Reichard, Tiana L. Sanders, Pabasara Weerarathne, James H. Meinkoth, Craig A. Miller, Ruth C. Scimeca, Consuelo Almazán

**Affiliations:** 1 Department of Veterinary Pathobiology, College of Veterinary Medicine, Oklahoma State University, Stillwater, OK 74078, USA; tiana.sanders@okstate.edu (T.L.S.); pabasara.weerarathne@okstate.edu (P.W.); james.meinkoth@okstate.edu (J.H.M.); craig.miller@okstate.edu (C.A.M.); ruth.scimeca@okstate.edu (R.C.S.); 2 Facultad de Ciencias Naturales, Universidad Autónoma de Querétaro, Avenida de las Ciencias S/N, Juriquilla, Querétaro 76230, Mexico

**Keywords:** *Cytauxzoon felis*, cytauxzoonosis, *Amblyomma americanum*, *Dermacentor variabilis*, North America

## Abstract

Cytauxzoonosis is an emerging tick-borne disease of domestic and wild felids produced by infection of *Cytauxzoon felis,* an apicomplexan protozoan similar to *Theileria* spp. Transmitted by *Amblyomma americanum*, lone star tick, and *Dermacentor variabilis*, American dog tick, infection of *C. felis* in cats is severe, characterized by depression, lethargy, fever, hemolytic crisis, icterus, and possibly death. Cytauxzoonosis occurs mainly in the southern, south-central, and mid-Atlantic United States in North America, in close association with the distribution and activity of tick vectors. Infection of *C. felis*, although severe, is no longer considered uniformly fatal, and unless moribund, every attempt to treat cytauxzoonosis cats should be made. Herein we review cytauxzoonosis, including its etiology, affected species, its life cycle and pathogenesis, clinical signs, diagnosis, and epidemiology, emphasizing clinical pathology findings in cats infected with this important emerging tick-borne disease in North and South America.

## 1. Introduction

Cytauxzoonosis is an emerging tick-borne disease produced by infection of *Cytauxzoon felis* (Piroplasmorida: Theileriidae), an apicomplexan protozoan that is transmitted by *Amblyomma americanum* and *Dermacentor variabilis* ticks to wild and domestic felids. Acute cytauxzoonosis in domestic cats is a severe clinical syndrome characterized by fever, inappetence, lethargy, depression, dehydration, dyspnea, hemolytic crisis, and icterus. Mortality can be high, but cats may also survive infection, and every attempt should be made to treat cats with cytauxzoonosis, especially if diagnosis is made early in the course of disease. If *C. felis*-infected cats survive acute disease, the cats will become chronic survivors, with only piroplasms present in erythrocytes. *Cytauxzoon felis* was first reported in four cats from southwestern Missouri that presented with anemia, icterus, dehydration, and fever [1]. Feline infectious anemia was originally suspected before schizonts consistent with *Cytauxzoon* spp. were noted in liver, lung, spleen, and lymph nodes from the cats at necropsy [1]. Later that same year, two cases of cytauxzoonosis were confirmed in domestic cats from central and southeastern Texas [2]. *Cytauxzoon felis* was established as the etiologic agent of cytauxzoonosis based on ultrastructural morphology, vertebrate host cell tropism, and mode of replication [3]. Acute cytauxzoonosis in domestic cats is colloquially referred to as bobcat fever, as cats with acute cytauxzoonosis are febrile at presentation and infected bobcats serve as wild animal reservoirs. Infections of *C. felis* are found primarily in the southern, south-central, and mid-Atlantic United States in North America, and it is likely that *C. felis* or closely related *Cytauxzoon* spp. are found in Brazil in South America [4,5,6,7]. This review is focused on the etiologic agent, affected species, life cycle and pathogenesis, clinical signs, diagnosis, and epizootiology of cytauxzoonosis, emphasizing the clinical pathology findings observed in cats infected with this important emerging disease in North America.

## 2. Etiologic Agent

*Cytauxzoon felis* is the only known piroplasm of domestic cats in the United States. Yabsley et al. [8] reported an unidentified *Babesia* sp. in Florida panthers (*Puma concolor coryi*); however, this unnamed piroplasm has not been identified in domestic cats. Numerous other piroplasms have been found in domestic and wild felids throughout the world [9,10,11], some of which were identified as *C. felis*. Identification of *C. felis* infection in domestic and wild cats outside of the Americas should be considered tentative until data can be provided that unequivocally confirm identification. Indeed, the taxonomy and definitive identification criteria for *Cytauxzoon* spp. are in need of elucidation [10]. Other species of *Cytauxzoon* in felids include: *C. manual* in Pallas’ cats (*Otocolobus manual*) [12], *C. europaeus* in European wildcats (*Felis sylvestris*) and Eurasian lynx (*Lynx lynx*) [11], *C. otrantorum* in European wildcats [11], and *C. banethi* in European wildcats [11]. The genus *Cytauxzoon* was erected in 1948 based on a piroplasm (*C. sylvicaprae*) found in a duiker (*Sylvicapra grimmia*) [13]. *Cytauxzoon* spp. are closely related to *Theileria* spp. sensu stricto and were differentiated based on vertebrate host leukocyte predilection. Schizogony for *Theileria* spp. occurs in lymphocytes with multiple fission in erythrocytes, whereas for *Cytauxzoon* spp., schizogony occurs in histiocytes with binary fission in erythrocytes [13,14]. However, differentiation between *Theileria* and *Cytauxzoon* based on vertebrate host leukocyte predilection may be inadequate and in need of reconsideration [15].

Gene sequence analysis of various *C. felis* isolates from the United States has demonstrated genetic variability across their range. Multiple studies have found genetic variation within internal transcriber spacer regions 1 and 2 (ITS1, ITS2) [16,17,18,19,20]. The ITSa genotype has been one of the most common genotypes reported, and while previously associated with higher survivability and found primarily in Arkansas, more recent evidence found it to occur in acute, fatal, and subclinical cases in domestic cats from Oklahoma, Arkansas, and Missouri [17]. Multiple genotypes have been reported to circulate within both domestic cats and wild felids. Pollard et al. [17] found ITS genotypes that had previously only been reported in wild felids also occuring in domestic cats, demonstrating the adaptivity of *C. felis.* Several studies have linked *C. felis* cytochrome *b* (*cytb*) genotypes with treatment success in response to administration of atovaquone and azithromycin (see below) [21,22]. However, Hartley et al. [23] reported a mutation in wild-type *C. felis cytb* methionine (M128). Post-treatment, *cytb* coded for isoleucine and valine at 2 and 4 months post-treatment despite repeated treatment with a higher dose of atovaquone in combination with azithromycin [23]. 

## 3. Affected Species

*Cytauxzoon felis* infects domestic and wild felids. Because of its similarity to *Theileria* spp. (e.g., *T. parva* and *T. annulata*), when *C. felis* was first identified, investigators were concerned that this parasite produced a severe infection, and fatal disease in cats may also have debilitating effects on production or other pet animals. Kier et al. [24] inoculated 33 different species of domestic, laboratory animal, and wildlife vertebrates with schizonts of *C. felis* to determine what impact the parasite may have on other animals and whether a laboratory animal model could be developed. The only animals that produced clear evidence of cytauxzoonosis were bobcats (*Lynx rufus*). 

### 3.1. Domestic Cats

Cases of cytauxzoonosis occur in cats throughout the southcentral, southeastern, and mid-Atlantic United States [25,26]. First reported in domestic cats from southwestern Missouri, cases of *C. felis* infection have since been reported from Alabama, Arkansas, Florida, Georgia, Illinois, Kentucky, Louisiana, Mississippi, North Carolina, Oklahoma, South Carolina, Tennessee, Texas, and Virginia (Table 1). Historically, infection of *C. felis* in domestic cats was considered highly fatal, with a very high mortality rate. However, cats surviving cytauxzoonosis were first noted around 2000 [19,25,27,28,29]. More recently, investigators have realized that there is a significant subpopulation of domestic cats that are subclinically infected with *C. felis*. Out of 902 blood samples of healthy, asymptomatic cats from Arkansas, Missouri, and Oklahoma, Rizzi et al. [30] demonstrated 15.5%, 12.9%, and 3.4%, respectively, were infected with *C. felis*. Similarly, in a study of 1,104 healthy cats in Kansas and 22 healthy cats in northwest Arkansas, 270 (25.8%) [31] and 4 (18.2%) [32], respectively, were infected with *C. felis*. Considering that (1) infection of *C. felis* is no longer considered uniformly fatal, (2) there is a significant subpopulation of domestic cats subclinically infected with *C. felis* found in close proximity to other domestic cats, and (3) transmission from cats subclinically infected with *C. felis* to naïve cats with *A. americanum* has been observed (46,47,50,52,53), it is likely these chronically infected cytauxzoonosis survivor cats are an important domestic reservoir of infection to other domestic cats.

### 3.2. Bobcats

Bobcats are the wild vertebrate reservoir of *C. felis.* Fatal infections of *C. felis* in bobcats have been reported [48,49]. However, the natural mortality rate of cytauxzoonosis in bobcats is not known, and given the high prevalence of *C. felis* infection in apparently healthy bobcats from enzootic areas (Table 2), the wild felid is considered a normal host for the parasite. Relatively few studies have been conducted to evaluate the occurrence of *C. felis* in bobcats. Nonetheless, the prevalence of *C. felis* infection is often high in areas where *A. americanum* and *D. variabilis* (see below) are present. Shock and colleagues [50] tested 696 bobcat spleens from 13 different states. The highest prevalence of *C. felis* in bobcats was reported in Missouri (79.5%), followed by North Carolina (62.5%), Oklahoma (65.0%), South Carolina (57.1%), Kentucky (55.4%), Florida (35.6%), Kansas (30.8%), Georgia (9.1%), and North Dakota (1.7%). *Cytauxzoon felis* was not detected in bobcats collected from Ohio, West Virginia, California, and Colorado. Birkenheuer et al. [51] surveyed bobcats in both North Carolina (where cytauxzoonosis is enzootic in domestic cats) and Pennsylvania (where cytauxzoonosis is not known to occur in domestic cats). They found bobcats in North Carolina had a significantly higher prevalence (33.0%) than Pennsylvania (7.3%). Shock et al. [50] also found a low prevalence of *C. felis* infection in bobcats from North Dakota (1.7%) where cytauxzoonosis is not known to occur in domestic cats. In southwestern Illinois, Zieman et al. [52] reported a prevalence of *C. felis* infection in bobcats of 70.6%. These same authors sequentially sampled five bobcats in their study area for 5 years and noted both that the wild felids were chronically infected with *C. felis* and that one of the cats became infected with a second strain of *C. felis* during the study [53]. More studies are needed to ascertain the transmission dynamics of *C. felis* spillover from bobcats in sylvatic ecosystems to domestic cats in urban settings. Additional surveys should also be conducted in bobcats to monitor changes in areas not previously recognized as enzootic for *C. felis* and to monitor changes locally in enzootic areas.

### 3.3. Puma and Florida Panthers

In addition to bobcats, infection of *C. felis* has been reported in pumas (also known as cougars or mountain lions [*Puma concolor*]) from North America [8,50,55,56] and South America [6] and Florida panthers (*Puma concolor coryi*; a subspecies of *P. concolor* distinguished for conservation efforts) from Florida [8,56,57]. Rotstein et al. [56] sampled pumas translocated from Texas to Florida as well as wild Florida panthers to estimate the impact of cytauxzoonosis on wild felids. In total they sampled 91 wild felids, finding that 11 out of 28 (39%) pumas and 22 out of 63 (35%) Florida panthers were infected with *C. felis*. While there were significant differences in blood parameters measured between the translocated pumas and the Florida panthers, the authors reported that biological differences in the blood values were not likely, as hematologic parameters measured were within expected ranges for healthy animals [56]. In addition, Rotstein et al. [56] noted the pumas translocated from Texas became infected with *C. felis* in Florida, as hemoparasites were not detected prior to arriving in Florida. It is probable that *Puma* spp. throughout North and South America are natural hosts for *C. felis* in enzootic areas; however, this hypothesis has yet to be tested.

### 3.4. Other Wild and Exotic Felids

Fatal cases of cytauxzoonosis have been reported from a captive Bengal tiger (*Panthera tigris*) at a zoo in Germany (14 months after importation of three bobcats from North America to the facility) [58], a captive-reared white tiger (*Panthera tigris*) in northern Florida [59], and captive-reared lions (*Panthera leo*) in Brazil [5]. Subclinical infections of *C. felis* have been reported in four captive tigers (*P. tigris*) in northwest Arkansas [60], six captive ocelots (*Leopardus pardalis*) in Brazil [6], and jaguars (*Panthera onca*) in Brazil [6,7]. Similar to bobcats and *Puma* spp., other wild felids native to North and South America are likely natural hosts for *C. felis* in enzootic areas.

## 4. Life Cycle and Pathogenesis

**Figure 1:**
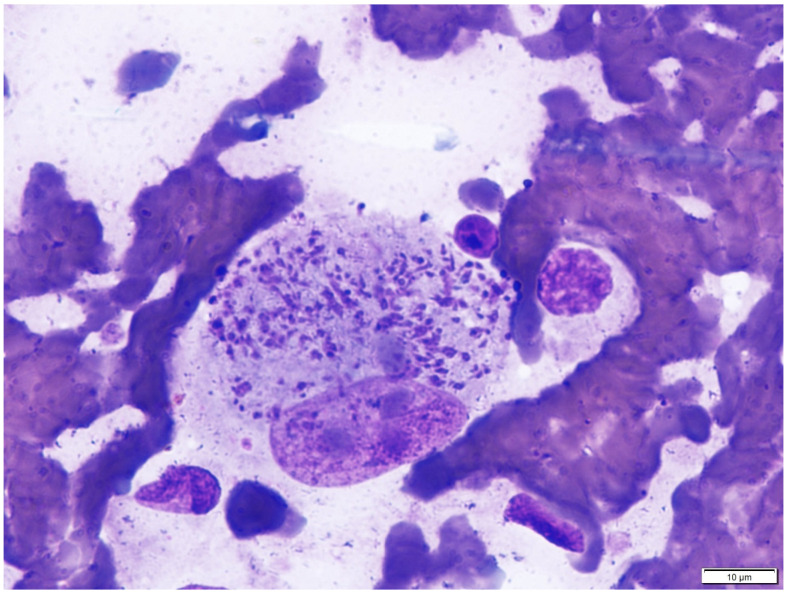
A schizont of *Cytauxzoon felis* from a stained impression smear of infected tissue. Schizonts range from 15–20 µm in diameter early in the course of infection up to 80–250 µm in diameter as disease progresses. As the disease progresses, the size and number of schizonts increases, leading to vascular occlusion.

The life cycle of *C. felis* occurs in two phases, one inside its tick vector (sexual) and the second inside the feline vertebrate host (asexual). Cats become infected with the transfer of *C. felis* sporozoites while an infected tick is feeding. Perinatal transmission of *C. felis* has been hypothesized but has not been demonstrated [61]. Ticks that have been experimentally demonstrated to transmit *C. felis* to cats include *A. americanum* adults [62,63,64,65], *A. americanum* nymphs [66], and *D. variabilis* adults [49,67]. *Amblyomma americanum* adults infected with *C. felis* need to be attached for a minimum of 36 to 48 h for cats to become infected [68,69]. Ingestion of *C. felis*-infected *A. americanum* adults is not a route of transmission to cats [69]. Inside the cat, sporozoites enter mononuclear cells, where they transform and undergo schizogony. During schizogony, the *C. felis*-infected host cell is transformed into a schizont (Figure 1) that can be found attached to the endothelium or within the lumina of veins and venules of all organs and tissues and within the interstitium of other tissues (e.g., spleen, lymph nodes) [70]. Schizonts are initially small in diameter (15 to 20 µm) and few until about day 12 post-infection. By day 19 post-infection, schizonts are larger (80 to 250 µm) and more numerous [70]. Schizogonous replication of *C. felis* results in distention and enlargement of the host cell schizont. Often referred to as megaschizonts, these cells can act like thrombi and occlude vessels. Vascular occlusion is a hallmark of acute cytauxzoonosis and is exhibited to some degree in all cases [71], resulting in multi-organ failure. Clinical signs of cytauxzoonosis begin 11–14 days after infected ticks begin feeding [62,63] and are considered a direct result of the *C. felis* schizogony process. 

**Figure 2:**
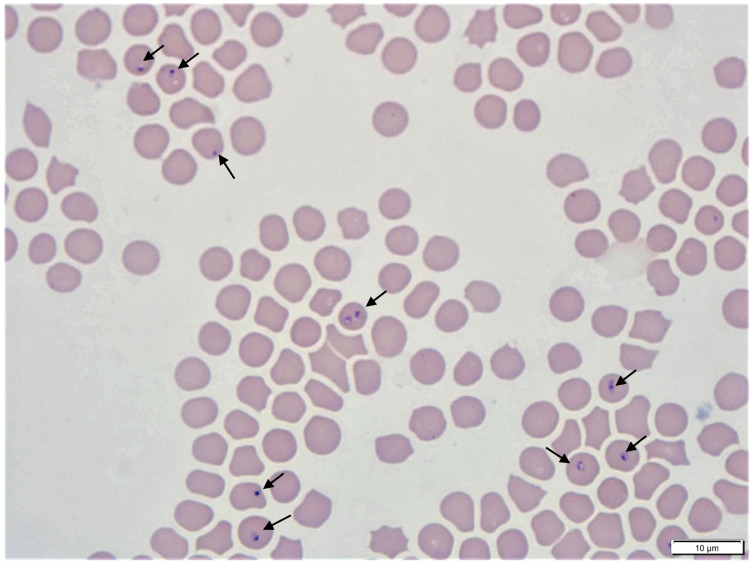
Piroplasms of *Cytauxzoon felis* in a stained thin-blood smear from an infected cat. Piroplasms of *C. felis* are morphologically variable but are generally pear-shaped, oval, ring-shaped, or anaplasmoid (arrows). They can occur singly, in pairs, or possibly in tetrads. Individual piroplasms measure 0.3–0.7 µm up to 1.0–2.2 µm in diameter or 0.8–1.0 µm in width by 1.5–2.0 µm in length depending on morphological form.

The schizogonous cycle of *C. felis* is considered limited [49], and this observation is supported in that schizonts are not found in cats that survive acute cytauxzoonosis [19,27,28,29,30,31]. Nevertheless, schizogony results in the formation of uninucleated merozoites that rupture from the schizonts, some of which are taken up by erythrocytes and become piroplasms (Figure 2). Piroplasms reproduce asexually within red blood cells through merogony, although many of the details of *C. felis* piroplasm multiplication and development are unknown and assumed from related *Theileria* spp. [72] At some point, *C. felis* piroplasms undergo gamogony, forming gametocytes in red blood cells, which at this time cannot be morphologically differentiated from merozoites. The gametocyte are what must be ingested by *A. americanum* or *D. variabilis* for the life cycle to continue. Once inside a tick, gametocytes metamorphose to gametes within the gut. Fertilization of piroplasm gametes results in the formation of a zygote that penetrates the peritrophic matrix and immediately invades the epithelial cells of the tick gut [72]. Inside the epithelial cells, the piroplasm zygote undergoes a meiotic division to form motile kinetes; once released in to hemolymph, they invade type II and III salivary glands [72]. In the salivary glands, they enlarge and transform into a sporont and then a sporoblast that is multinucleated [72]. Formation of the sporoblast is associated with hypertrophy of infected salivary glands for *C. felis* and other closely related piroplasms [72]. Sporogony occurs asynchronously, providing a continuous release of sporozoites into tick saliva and to the feline hosts while infected ticks are feeding [72]. 

**Figure 3:**
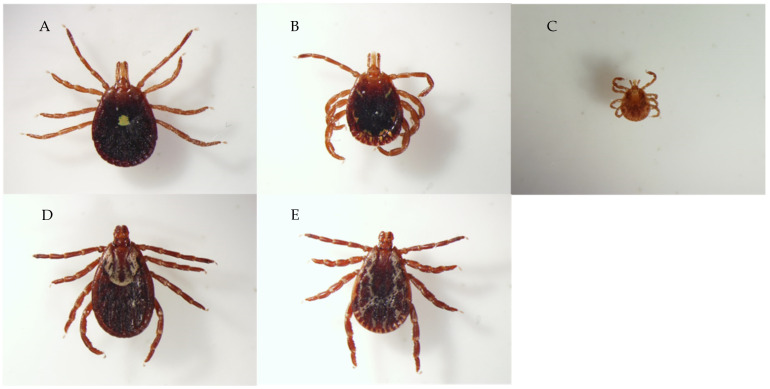
Ticks that have been experimentally demonstrated to act as vector for *Cytauxzoon felis* by feeding infected ticks on domestic cats or bobcats include: (**A**) *Amblyomma americanum* female, (**B**) *A. americanum* male, (**C**) *A. americanum* nymph, (**D**) *Dermacentor variabilis* female, (**E**) *D. variabilis* male. Images courtesy of Megan Lineberry, National Center for Veterinary Parasitology. All images 1.6× magnification.

Initial attempts at transmitting *C. felis* through ticks were unsuccessful [73] (as reported in [3]). However, Blouin et al. [67] successfully transmitted *C. felis* by acquisition-feeding *D. variabilis* nymphs on an infected bobcat that was splenectomized, and then transmission-feeding the adult ticks (Figure 3) on two splenectomized domestic cats. Blouin et al. [49] subsequently confirmed the ability of *D. variabilis* to transmit *C. felis* by acquisition-feeding *D. variabilis* nymphs on another splenectomized bobcat and transmission-feeding the adult ticks on two spleen-intact bobcats. Reichard et al. [63] acquisition-fed nymphs of *A. americanum*, *D. variabilis*, *Ixodes scapularis*, and *Rhipicephalus sanguineus* on a naturally infected, *C. felis* survivor domestic cat. Once the nymphs had fed to repletion, they were molted to adults, and then ticks of each species were transmission-fed on individual cats. The cat infested with *A. americanum* adults (Figure 3) was the only one that became infected with *C. felis*. Reichard et al. [62] confirmed the ability of *A. americanum* to act as a vector for *C. felis* by acquisition-feeding nymphs of *A. americanum* and *D. variabilis* simultaneously on a subclinically infected *C. felis* survivor cat and subsequently transmission-feeding adults of each tick species on four domestic cats. All four of the *A. americanum* transmission-fed cats became infected with *C. felis*, whereas none of the *D. variabilis*-fed cats became infected. Allen et al. [66] acquisition-fed *A. americanum* and *D. variabilis* larvae on a parasitemic cytauxzoonosis survivor cat and then transmission-fed nymphs of those ticks on each of three cats. Only the three cats infected with *A. americanum* nymphs (Figure 3) became infected with *C. felis*. 

Surveys on the occurrence and prevalence of *C. felis* in ticks are limited (Table 3). Bondy et al. [74] amplified DNA of *C. felis* in partially engorged *A. americanum* nymphs recovered from a cat that died of acute cytauxzoonosis. Reichard et al. [62] reported minimum infection rates of *C. felis* in unengorged *A. americanum* females at 1.5%, *A. americanum* males at 0.5%, and *A. americanum* nymphs at 0.8%, and no infections in *D. variabilis* females and males from north-central Oklahoma. Shock et al. [75] tested ticks from Georgia, Kentucky, Pennsylvania, Tennessee, and Texas for *C. felis* infection, and detected *C. felis* only in *D. variabilis* from Tennessee and Georgia. Infection of *C. felis* in *A. americanum* was not detected from those states (Table 3). Zieman et al. [52] collected *A. americanum* and *D. variabilis* from an enzootic area where 70.4% of bobcats were infected with *C. felis*. They found 15.4% of *A. americanum* and 15.8% of *D. variabilis* were infected with *C. felis* in southern Illinois [52]. 

*Amblyomma americanum* is considered the primary vector of *C. felis* in the United States due to the overlap in distribution and abundance of lone star ticks in the southern Unites States with that of cytauxzoonosis cases [51], corresponding seasonal activity of *A. americanum* and occurrence of clinical cases in domestic cats [47], and host preference of lone star ticks compared to American dog ticks and the likelihood of those ticks feeding on cats [76,77]. Additionally, the three studies that have been performed comparing the competency of *A. americanum* and *D. variabilis* in the transmission of *C. felis* to domestic cats [62,63,66] demonstrated transmission only with lone star ticks. Nevertheless, it is evident that *D. variabilis* can be involved in the transmission of *C. felis*, as it has been demonstrated experimentally [49,67], *C. felis* has been found in questing American dog ticks [52,75], and *C. felis* has been found in bobcats outside the range of lone star ticks but in areas where American dog ticks occur [51,75]. Considerably more research needs to be performed to determine the transmission dynamics of *C. felis* to domestic cats and the vector competence of *A. americanum*, *D. variabilis*, and possibly other ticks throughout the ranges of the parasites. As we are currently appreciating considerable change in our comprehension in the distribution of *D. variabilis* [78] and expansion in the range of *A. americanum* [79,80], it will become ever more important to understand the transmission dynamics, the role of these tick vectors (and possibly others), and the risk of cytauxzoonosis to cats in areas not currently recognized as enzootic.

It is still unknown what ticks transmit *C. felis* or the *C. felis*-like organism(s) in South America. *Amblyomma cajennense* were found in the habitat of captive lions that died of cytauxzoonosis in Rio de Janeiro [5]. However, these ticks could not be definitively linked to transmission, as no *C. felis* were found in hemolymph nor histological sections of the ticks. Currently, 44 species of hard ticks are recognized in Brazil: 30 species of *Amblyomma*, 1 species of *Dermacentor*, 3 species of *Haemaphysalis*, 8 species of *Ixodes*, and 2 species of *Rhipicephalus* [81].

**Figure 4:**
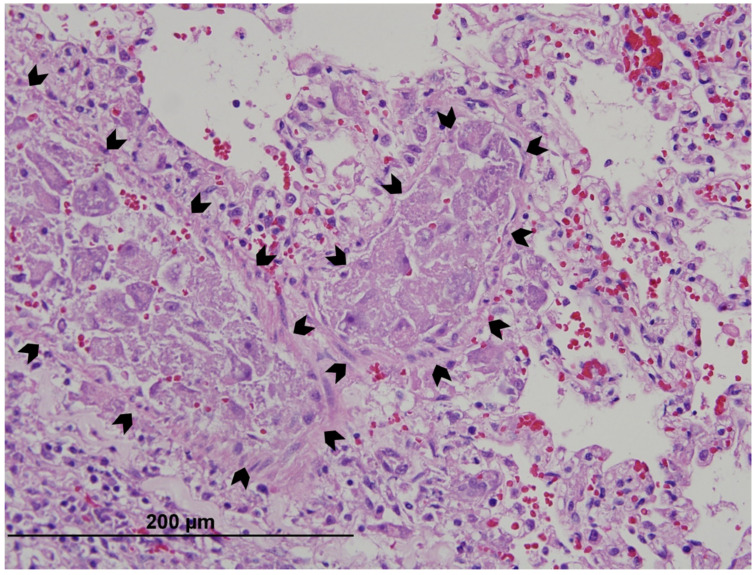
Pulmonary vessels (outlined with chevrons) occluded by schizonts of *Cytauxzoon felis*. As schizonts replicate, they become larger and more numerous in the veins and sinusoids of many tissues. If infection is severe, vessels in any parenchymatous organ may contain schizonts.

Pathogenesis of cytauxzoonosis is largely attributed to schizogony of *C. felis* in histiocytes. These cells accumulate in the veins and sinusoids of many tissues [25]. In severe cases, schizonts of *C. felis* may occlude the lumen (Figure 4) of these vessels [70]. Thrombosis of affected vessels is common, and histological changes consistent with ischemia are seen in many tissues, including the brain and heart [82]. The lungs, spleen, and liver are usually the most severely affected organs, but most any parenchymatous organs can be involved [25]. Evaluation of the pulmonary histopathology of 148 *C. felis* infections from Oklahoma showed moderate interstitial pneumonia, mild alveolar macrophage involvement, mild intra-alveolar hemorrhage, and moderate to severe vascular occlusion, with pulmonary edema common [71]. A histopathology review of eight cases of *C. felis* infection from Georgia showed the presence of intravascular schizont-laden macrophages in leptomeningeal and parenchymal arterioles and venules, along with occlusion of small capillaries throughout the gray and white matter and choroid plexus [83].

## 5. Clinical Signs

**Figure 5:**
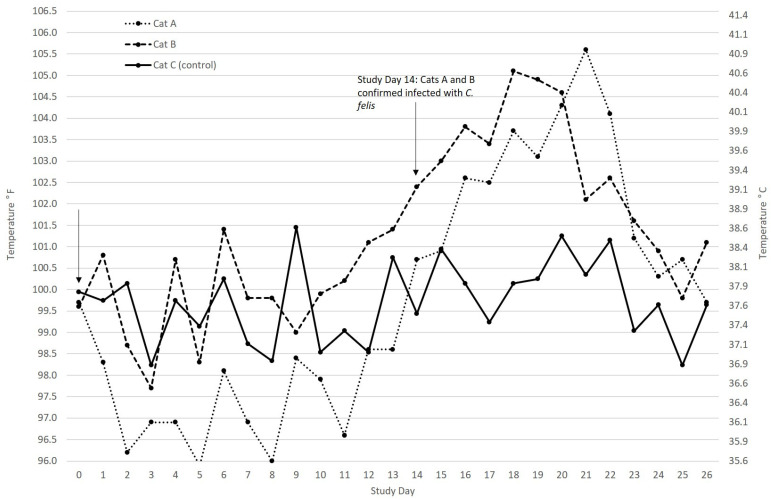
Temperature profile of two cats (A and B) with cytauxzoonosis compared to a control cat (C) that was not infected. Onset of cytauxzoonosis typically begins 11–14 days after being bitten by a *C. felis*-infected tick. Fever can peak around 40.6–41.1 °C (105.0–106.0 °F). Once cats begin to recover, they can become hypothermic before the temperature returns to normal or they become moribund.

**Figure 6:**
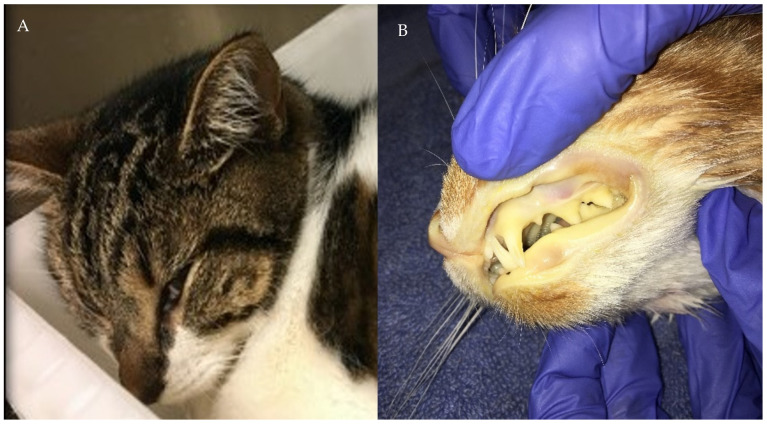
Cats that developed cytauxzoonosis from infection with *Cytauxzoon felis*. (**A**) Depression and lethargy are the first clinical signs owners notice with cats infected with *C. felis*. This cat would be dehydrated and febrile at presentation. (**B**) Cats severely affected by cytauxzoonosis can become anemic and icteric, the degree of which can be highly variable among *C. felis* infected cats.

Infection of *C. felis* in domestic cats is severe. Cats present with fever (Figure 5), inappetence, lethargy, depression, dehydration, dyspnea, hemolytic crisis, and possibly icterus (Figure 6). Before 2000, cytauxzoonosis was considered a uniformly fatal disease. However, that is no longer the case, and a considerable number of cats have been documented to survive acute cytauxzoonosis [19,25,26,27,28,29,30,31,32]. Additionally, current treatment strategies (see below) improve the likelihood of survival to discharge by a factor of over 7 [84]. Unless a cat infected with *C. felis* is moribund at presentation, every attempt should be made to treat and recover the cat. Cats become febrile approximately 11–14 days after being bitten by a *C. felis*-infected tick. A typical case of cytauxzoonosis as seen at presentation with CBC and chemistry panel is provided in Table S1. 

Cats displaying signs of cytauxzoonosis will likely have a low white blood cell count (leukopenia), characterized by low neutrophils with a left shift and toxic change. Cats will be thrombocytopenic and possibly anemic. Upon examination of blood smears, piroplasms of *C. felis* may not be present during acute disease or if cats are being treated with the recommended therapy (see below). Cats initially have a non-regenerative anemia, but if they survive, schizogonous replication of *C. felis* they will become regenerative at some point. The anemia is attributable to both hemolysis (not increase in bilirubin) and bone marrow suppression. Large granular lymphocytes may be normal or increased. However, if large granular lymphocytes are present in leukopenic or neutropenic cats, the index of suspicion for *C. felis* infection is high. Cats that survive acute cytauxzoonosis become persistently infected [84] and are considered life-long carriers of *C. felis*.

The current recommended treatment for cytauxzoonosis includes a combination of atovaquone (15 mg/kg PO q8H) and azithromycin (10 mg/kg PO q24h) [84]. Use of diminazene diaceturate was hypothesized to clear *C. felis* subclinically infected carrier cats, but this treatment was not effective, and adverse side effects were common [85]. Administration of atovaquone and azithromycin therapy combined with aggressive supportive and nursing care [26] resulted in a 60% survival rate, and treated cats were 7.2 times more likely to survive to discharge [84]. Recommended supportive care measures to consider, depending on specifics of the case, include judicious intravenous crystalloid fluid therapy, heparin to prevent disseminated intravascular coagulation, analgesic therapy, antiemetics, red blood cell transfusion, fresh or frozen plasma, oxygen supplementation, therapeutic thoracocentesis, and nutritional support [26]. Interestingly, administration of antipyretic agents may be contraindicated but deserves further evaluation [26].

Bioinformatic analysis of the *C. felis* genome has been used to predict a candidate vaccine for cytauxzoonosis [86]. However, a vaccine for cytauxzoonosis has not yet become commercially available. Disease prevention currently relies on administration of acaricides to cats to control tick feeding. Two products, approved for use on cats in the United States, have demonstrated efficacy for blocking the transmission of *C. felis* to cats by preventing or interrupting feeding of infected *A. americanum*: Seresto (imidacloprid and flumethrin collar) [65] and Revolution Plus (selamectin and sarolaner topical solution) [64]. 

## 6. Diagnosis 

Definitive diagnosis of cytauxzoonosis is based on observation of *C. felis* in infected tissue or by detecting parasites through a molecular-based method, typically PCR. The most widely used but least-sensitive method for diagnosing *C. felis* infection is microscopic observation of Wright–Giemsa stained thin-blood smears for piroplasms of *C. felis* in erythrocytes (Figure 2). Piroplasms of *C. felis* are piriform (i.e., pear-shaped) but can also be found in ring, oval, or anaplasmoid forms, occurring as singles, pairs, or possibly tetrads (i.e., maltese crosses), and measure 0.3–0.7 µm up to 1.0–2.2 µm in diameter or 0.8–1.0 µm in width by 1.5–2.0 µm in length depending on morphological form [70]. Clinical signs of cytauxzoonosis may precede the presence of *C. felis* piroplasms in erythrocytes by several days or more [26]. Cats that survive acute cytauxzoonosis will develop a low-level parasitemia in ≤1% of erythrocytes. While definitive evidence has not been provided, cats that survive cytauxzoonosis are considered life-long carriers of *C. felis* and can be reservoirs of infection if not provided with effective tick prevention. Schizonts of *C. felis* precede the production of piroplasms in erythrocytes and can be observed in fine needle aspirates of infected organs (e.g., spleen, lymph nodes), histopathology, or impression smears. Schizonts range from 15–20 µm in diameter early in the course of infection up to 80–250 µm in diameter as disease progresses (Figure 1) [70]. As cats become more ill and the size of schizonts increases, so does the number of schizonts, which leads to vascular occlusion. Despite substantial pulmonary pathology due to *C. felis* infection, pathognomonic lesions of acute cytauxzoonosis are not evident on thoracic radiographs [87]. 

Polymerase chain reactions using primers that amplify specific genetic segments of *C. felis* (Table 4) are the most widely used molecular methods employed for diagnosing *C. felis* infection. These molecular methods are considerably more sensitive and specific compared to light microscopy but are more time-consuming and costly. A patient-side assay that can aid veterinary practitioners in diagnosis cytauxzoonosis would be considerably advantageous and would allow initiation of treatment early in the course of disease. Different genetic targets used for *C. felis* diagnosis include 18S rRNA [19,63,74,88], internal transcribed spacer 1 (ITS1) [19], ITS 2 [19,28,63], cytochrome b (*cytb*) [89], and cytochrome c oxidase subunit III (*cox3*) [89,90]. Of the PCR methods available, digital droplet PCR (ddPCR) is the most sensitive assay, detecting as little as 0.175 copies/µL, and can provide an absolute quantification of parasite load over time while requiring only a small quantity of DNA (as little as 0.0000231 ng DNA/reaction) [90]. Other sensitive quantitative PCR methods are nested-PCR targeting 18S rRNA [63,74] and real-time PCR targeting ITS2 region [63]. In addition to these molecular methods, in situ hybridization has been used to visualize and confirm the *C. felis* in tissue samples [91].

## 7. Epizootiology 

Presentation of cytauxzoonosis cases to veterinary clinics follows a bimodal pattern that is related to the seasonal activity of ticks [47]. Tick activity is dependent on environmental factors such as temperature range, precipitation, and humidity [92]. Peak activity of adult and nymphal *A. americanum* occurs from April to June and August to September, respectively [47,50], but may differ across geographical regions. Other environmental factors such as low-density residential areas, wooded habitat, and proximity to natural or unmanaged areas [47] pose a higher risk of *C. felis* infection to domestic cats. Not only do these environmental factors suit the tick vectors, but wooded habitats and edge habitats also provide suitable conditions for bobcats [47]. However, the bimodal pattern of clinical cases correspond with seasonal fluctuations of infected tick vectors more than activity of bobcats [47], indicating that clinicians should be aware of the seasonal activity of tick vectors in their area to best guide their client prevention, treatment, and control protocols. 

In addition to environmental risk factors, age, sex, and lifestyle may influence the risk of cytauxzoonosis to domestic cats. More clinical cases have been diagnosed in young cats from 1–4 years of age [44,93]. There are several possible explanations of why young cats may contribute to higher clinical cases. Young cats may have a greater drive to explore territory, risking exposure to tick vectors; clients may bring in younger cats with acute illness more often than older cats, in whom illness may be contributed to by old age; and older cats that have recovered from previous infection with *C. felis* may be asymptomatic carriers [93]. Multiple studies have found that young, male cats were over-represented when diagnosing acute cytauxzoonosis as well [44,93]. Cats that spend most of their lives outdoors have a significantly higher risk of becoming infected with *C. felis* due to tick exposure. A study in Kansas found 29.6% of feral cats were positive for *C. felis* compared to 25.4% of owned cats and 21.8% of rescue cats [93]. These feral cats spent their entire lives outdoors, greatly increasing their risk of tick exposure. 

Initially, infection of *C. felis* was considered almost 100% fatal. In fact, many practitioners would euthanize upon a definitive cytauxzoonosis diagnosis. However, several studies conducted in disparate geographical areas have documented the presence of subclinical, chronically *C. felis*-infected domestic cats [27,28,29,30]. Prior to the realization that domestic cats were surviving infection, bobcats were considered the only vertebrate reservoir. With free-roaming and feral domestic cats being found in closer proximity to client-owned domestic cats, and knowing that tick transmission can occur from a subclinically infected carrier domestic cat to a naïve domestic cat [62,63,64,65,66,69], it is likely that *C. felis*-infected subclinical carrier domestic cats with access to the outdoors are domestic vertebrate reservoirs, along with infected bobcats being wild vertebrate reservoirs.

## Figures and Tables

**Table 1:** Reports and surveys of domestic cats for *Cytauxzoon felis* infection in North America.

State	No. of Cats Tested	No. of Cats Infected	Prevalence; 95% Confidence Interval	Sample Period	Sample Type	Test Method	Reference
Alabama	NA	NA	NA	NA	NA	NA	[33]
Arkansas	NA	NA	NA	NA	NA	NA	[34]
	NA	18	NA	1997–1998	blood	microscopy and PCR for 18S	[29]
	NA	3	NA	NA	blood	microscopy and PCR for ITS 1&2	[28]
	NA	57	NA	2005–2007	blood	microscopy and PCR for ITS 1&2	[18]
	43	18	41.9%; 28.4–56.7%	NA	blood	microscopy and PCR for ITS 1&2	[19]
	NA	12	NA	1998–2011	blood or other tissue	microscopy	[35]
	161	25	15.5%; 10.7–22.0%	2008–2012	blood	PCR for18S	[30]
	22	4	18.2%; 6.7–39.1%	2020–2021	blood	PCR for cox3	[32]
Florida	494	1	0.2%; <0.1–1.3%	1999–2000	blood	PCR for18S	[27]
Georgia	NA	NA	NA	NA	NA	NA	[34]
	NA	9	NA	NA	blood or other tissue	microscopy	[36]
	NA	31	NA	2005–2007	blood	microscopy and PCR for ITS 1&2	[18]
	46	9	19.6%; 10.4–33.4%	NA	blood	microscopy and PCR for ITS 1&2	[19]
Illinois	59536	12	NA	2003–2012	blood and spleen	microscopy and PCR for ITS 1&2	[37]
Indiana	NA	NA	NA	NA	NA	NA	[38]
Iowa	292	0	0.0%; 0.0–1.3%	2012–2014	blood	PCR for18S	[39]
Kansas	NA	1	NA	NA	lung and liver	microscopy	[40]
	1104	270	25.8%; 22.0–27.1%	2018–2019	blood	PCR: cox3	[31]
Kentucky	NA	1	NA	NA	brain, heart, lung, intestine, spleen, lymph node, and kidney	microscopy	[41]
	NA	56	NA	2001–2011	blood or other tissue	microscopy	[42]
Louisiana	NA	1	NA	NA	blood and other tissue	microscopy	[43]
Mississippi	NA	NA	NA	NA	NA	NA	[33]
Missouri	NA	4	NA	1973–1975	liver, lung, spleen, and lymph nodes	microscopy	[1]
	NA	68	NA	1998–2011	blood or other tissue	microscopy	[35]
	62	8	12.9%; 6.4–23.7%	2008–2012	blood	PCR for18S	[30]
North Carolina	NA	28	NA	1998–2004	blood or other tissue	microscopy	[44]
	392	0	0.0%; 0.0–1.2%	1999–2000	Blood	PCR for18S	[27]
Oklahoma	NA	2	NA	1984	blood or other tissue	microscopy	[45]
	NA	8	NA	1985–1992	blood or other tissue	microscopy	[46]
	NA	18	NA	1997–1998	blood	microscopy and PCR for 18S	[29]
	NA	232	NA	1995–2006	blood or other tissue	microscopy	[47]
	NA	130	NA	1998–2011	blood or other tissue	microscopy	[35]
	679	23	3.4%; 2.2–5.1%	2008–2012	blood	PCR for18S	[30]
	380	3	0.79%; 0.2–2.4%	2012–2014	blood	PCR for18S	[39]
South Carolina	NA	3	NA	1998–2004	blood or other tissue	microscopy	[44]
Tennessee	75	1	1.3%; <0.1–7.9%	2006	blood	PCR for18S	[27]
Texas	NA	2	NA	NA	tissue	microscopy	[2]
Virginia	NA	3	NA	1998–2004	blood or other tissue	microscopy	[44]

NA, not assessed.

**Table 2:** Reports and surveys of bobcats (*Lynx rufus*) for *Cytauxzoon felis* infection in North America.

State	No. of Bobcats Tested	No. of Bobcats Infected	Prevalence; 95% Confidence Interval	Sampling Period	Sample Tested	Test Method	Reference
Arkansas	6	NA	NA	NA	spleen	real-time PCR for 18S; PCR for ITS1 and ITS2	[19]
California	26	0	0.0%; 0.0–15.2%	1999–2010	blood or spleen	nested PCR for ITS1	[50]
Colorado	67	0	0.0%; 0.0–6.5%	1999–2010	blood or spleen	nested PCR for ITS1	[50]
Florida	45	16	35.6%; 23.2–50.2%	1999–2010	blood or spleen	nested PCR for ITS1	[50]
	54	NA	NA	NA	spleen	real-time PCR for 18S; PCR for ITS1 and ITS2	[19]
Georgia	143	13	9.1%; 5.3–15.1%	1999–2010	blood or spleen	nested PCR for ITS1	[50]
	73	NA	NA	NA	spleen	real-time PCR for 18S; PCR for ITS1 and ITS2	[19]
Illinois	125	88	70.4%; 61.9–77.7%	2003–2015	blood or spleen	nested PCR for 18S	[52]
Kansas	39	12	30.8%; 18.5–46.5%	1999–2010	blood or spleen	nested PCR for ITS1	[50]
	1	1	NA	2000		microscopy	[48]
Kentucky	74	41	55.4%; 44.1–66.2%	1999–2010	blood or spleen	nested PCR for ITS1	[50]
Missouri	39	31	79.5%; 64.2–89.5%	1999–2010	blood or spleen	nested PCR for ITS1	[50]
North Carolina	30	10	33.3%; 19.3–51.3%	2004, 2005, 2006	blood	PCR for18S	[51]
	8	5	62.5%; 30.4–86.5%	1999–2010	blood or spleen	nested PCR for ITS1	[50]
North Dakota	172	3	1.7%; 0.4–5.2%	1999–2010	blood or spleen	nested PCR for ITS1	[50]
Pennsylvania	69	5	7.3%; 2.8–16.2%	2002	blood	PCR for 18S	[51]
Ohio	19	0	0.0%; 0.0–19.8%	1999–2010	blood	nested PCR for ITS1	[50]
Oklahoma	10	NA	NA	1982–1984	blood, liver, spleen, lung, or lymph nodes	microscopy	[49]
	20	13	65.0%; 43.2–82.0	1999–2010	blood or spleen	nested PCR for ITS1	[50]
	26	13	50.0%; 32.1–67.9%	~1982	blood	microscopy	[54]
South Carolina	7	4	57.1%; 25.0–84.3%	1999–2010	blood or spleen	nested PCR for ITS1	[50]
West Virginia	37	0	0.0%; 0.0–11.2%	1999–2010	blood or spleen	nested PCR for ITS1	[50]

**Table 3:** Reports and surveys of ticks for *Cytauxzoon felis* infection in North America.

State	Tick Species	Tick Life Stage	No. of Ticks or Tick Pools Tested	No. of Ticks or Tick Pools Infected	Prevalence or Minimum Infection Rate (95% Confidence Interval)	Reference
Georgia	*Amblyomma americanum*	NR *	340	0	0.0% (0.0–1.4%)	[75]
	*Dermacentor variabilis*	NR	125	1	0.8% (<0.1–4.8%)	
	*Amblyomma maculatum*	NR	16	0	0.0% (0.0–22.7%)	
	*Ixodes scapularis*	NR	3	0	0.0% (0.0–61.8%)	
	*Amblyomma* spp.	NR	2	0	0.0% (0.0–71.0%)	
Illinois	*A. americanum*	female	57	8	14.0% (7.0–25.6%)	[52]
		male	60	10	16.7% (9.1–28.3%)	
	*D. variabilis*	female	51	10	19.6% (10.8–32.6%)	
		male	50	6	12.0% (5.3–24.1%)	
Kentucky	*A. americanum*	NR	61	0	0.0% (0.0–7.1%)	[75]
	*D. variabilis*	NR	42	0	0.0% (0.0–10.0%)	
Missouri	*A. americanum*	adult	210	0	0.0% (0.0–2.2%)	[74]
		nymph	16	3 †	18.8% (5.8–43.8%) †	
	*D. variabilis*	adult	79	0	0.0% (0.0–5.6%)	
	*Rhipicephalus sanguineus*	adult	35	0	0.0% (0.0–11.8%)	
Oklahoma	*A. americanum*	female	49	3	1.9% (1.5–17.2%)	[62]
		male	46	1	0.7% (<0.1–12.4%)	
		nymph	80	3	0.9% (0.8–10.9%)	
	*D. variabilis*	female	23	0	0.0% (0.0–16.9%)	
		male	28	0	0.0% (0.0–14.3%)	
Pennsylvania	*Ixodes scapularis*	NR	1	0	0.0% (0.0–83.3%)	[75]
Tennessee	*A. americanum*	NR	184	0	0.0% (0.0–2.5%)	[75]
	*D. variabilis*	NR	442	8	1.8% (0.9–3.6%)	
Texas	*A. americanum*	NR	158	0	0.0% (0.0–2.9%)	[75]
	*D. variabilis*	NR	93	0	0.0% (0.0–4.8%)	
	*Amblyomma cajennense*	NR	99	0	0.0% (0.0–4.5%)	
	*Amblyomma* spp.	NR	64	0	0.0% (0.0–6.8%)	
	*Ixodes woodi*	NR	1	0	0.0% (0.0–83.3%)	

* NR: Not reported. † Ticks were recovered off a *Cytauxzoon felis* infected cat and cannot ascertain if ticks were infected or if they tested positive because of host blood.

**Table 4:** Summary of molecular methods used for diagnosing *Cytauxzoon felis* in infected tissues.

Method *	Host Sample	Gene Target	Amplicon Length	Primers	Reference
PCR	Blood, other tissues	18S	284 bp	F: 5′-GCGAATCGCATTGCTTTATGCT-3′ R: 5′-CAATTGATACTCCGGAAAGAG-3′	[88]
PCR	Blood, other tissues	cytb	1203 bp	F: 5′-AGGATACAGGGCTATAACCAAC-3′ R: 5′-GTACTCTGGCTATGTCAATTTCTAC-3′	[22]
PCR	Blood, other tissues	ITS2, partial 5.8S and 28S	431 bp	F: 5′-TGAACGTATTAGACACACCACCT-3′ R: 5′-TCCTCCCGCTTCACTCGCCG-3′	[28]
PCR	Blood, other tissues	18S	82 bp	F: 5′-TGC ATC ATT TAT ATT CCT TAA TCG-3′ R: 5′-CAA TCT GGA TAA TCA TAC CGA AA-3′	[19]
PCR	Blood, other tissues	ITS1	651 bp (domestic cats)	F: 5′-CGA TCG AGT GAT CCG GTG AAT TA-3′ R: 5′-GCT GCG TCC TTC ATC GAT GTG-3′	[19]
PCR	Blood, other tissues		746 bp (bobcats)	F: 5′-CGA TCG AGT GAT CCG GTG AAT TA-3′ R: 5′-GGA GTA CCA CAT GCA AGC AG-3′	
PCR	Blood, other tissues	ITS2	475 bp	F: 5′-AGC GAA TTG CGA TAA GCA TT 3′ R: 5′-TCA GCC GTT ACT AGG AGA-3′	[19]
PCR	Blood, other tissues	18S	Primary—700 bp	F: 5′-ACCTGGTTGATCCTGCCAGTAGTCATATGCTTG-3′ R5′-TCACCAGAAAAAGCCACAAC-3′	[74]
			Nested—289 bp	F: 5′-TCGCATTGCTTTATGCTGGCGATG-3′ R: 5′-GCCCTCCAATTGATACTCCGGAAA-3′	[63]
ddPCR	Blood, other tissues	cox3	118 bp	F: 5′-CTACACTCTTTACACGTTTGTG -3′ R: 5′-AGGAGTATACTGGCATTTCG -3′	[90]
ISH	Formalin-fixed, paraffin-embedded tissues	16S-like	600 bp	F: 5′-CATGTCTTAGTATAAGCTTTTATACAGAA-3′ R: 5′-AACGCTGCGGAAGCGAGATTAATGACAAGGCAG-3′	[91]

* Abbreviations, PCR: polymerase chain reaction; ddPCR: droplet digital PCR; ISH: in situ hybridization.

## Supplementary Materials

The following are available online at https://www.mdpi.com/article/10.3390/pathogens10091170/s1. Table S1: Results of complete blood count (CBC), and serum chemistry profile (SCP) obtained from a typical case of acute cytauxzoonosis in a cat presented to a veterinary clinic in an enzootic region.
