# Supplementary material for: Cytauxzoonosis in North America
Source: Pathogens. 2021 Sep 10;10(9):1170. doi: 10.3390/pathogens10091170 (PMC8469551; doi:10.3390/pathogens10091170)
Supplement: Supplementary file 1 [file pathogens-10-01170-s001.zip › pathogens-1351116-supplementary.pdf]

Typical Case of Cytauxzoonosis

History: A 7-month-old neutered male cat from Payne County in north-central Oklahoma with 2-day history of lethargy, depression, inappetence, dehydration, and icterus. Owner suspected infection with *Cytauxzoon felis* and reported a history of five other cats from their property, previously infected.

Table S1 Results of complete blood count (CBC) obtained from a typical case of acute cytauxzoonosis in a cat presented to a veterinary clinic in an enzootic region.

Test	Results (Unit)	Reference Values (Units)	Comments
WBC	1.0 x 10^3 /μL	3.5–16.0 x 10^3 /μL	Low
RBC	6.3 x 10^6 /μL	5.92–9.93 x 10^6 /μL	
HGB	8.3 g/dL	9.3–15.9 g/dL	Low
HCT	25%	29–48%	Low
MCV	39 fL	37–61 fL	
MCH	13.1 pg	11–21 pg	
MCHC	34 g/dL	30–38 g/dL	
Poikilocytosis	Moderate		
Blood parasites			See clinical pathology review below
Platelet count	7 x 10^3 /μL	200–500 × 10^3 /μL	Low
Platelet estimate	Decreased		
Neutrophils	32%	35–75%	Low
Bands	16%	0–3%	High
Lymphocytes	44%	20–45%	
Monocytes	8%	1–4%	High
Eosinophils	0%	2–12%	Low
Basophils	0%	0–1%	
Absolute neutrophils	320 /μL	2500–8500 /μL	Low
Absolute bands	160 /μL	0–150 /μL	High
Absolute lymphocytes	440 /μL	1200–8000 /μL	Low
Absolute monocytes	80 /μL	0–600 /μL	
Absolute eosinophils	0 /μL	0–1000 /μL	

Clinical Pathology Review: Erythrocyte density appeared mildly decreased, consistent with the reported hematocrit. Lack of polychromasia and findings on the ADVIA erythrocyte cytogram indicated a non-regenerative anemia. Abundant 2–3 μm signet-ring-shaped and elongated small protozoal piroplasms (i.e. *Cytauxzoon felis*) were identified with erythrocyte cytoplasm. Low numbers of ghost cells were present.

Leukocyte density and differential were confirmed. A marked leukopenia was present due to a marked neutropenia. While only rare neutrophils were present in the blood, a significant number of these exhibited band morphology. Toxic change was mild (cytoplasmic basophilia and foaminess).

A manual platelet estimate revealed an average of 1 platelet per 100x field, yielding an estimate of 15,000–30,000/μL. Clumps of fibrin were noted at the feathered edge, but no true platelet clumps were found. Overall, platelets were estimated to be markedly decreased in number.

Table S1. Serum chemistry profile (SCP) obtained from a typical case of acute cytauxzoonosis in a cat presented to a veterinary clinic in an enzootic region.

Test	Results (Unit)	Reference Values (Units)	Comments
Total Protein	5 g/dL	5.2–8.8 g/dL	Low
Albumin	2.6 g/dL	2.5–3.9 g/dL	
Globulin	2.4 g/dL	2.3–5.3 g/dL	
A/G Ratio	1.1	0.35–1.5	
AST (SGOT)	86 IU/L	10–100 IU/L	
ALT (SGPT)	47 IU/L	10–100 IU/L	
Alk Phosphatase	5 IU/L	6–102 IU/L	Low
GGT	1 IU/L	1–10 IU/L	
Total Bilirubin	7.0 mg/dL	0.1–0.4 mg/dL	High
BUN	89 mg/dL	14–36 mg/dL	High
Creatine	1.7 mg/dL	0.6–2.4 mg/dL	
BUN/CREAT Ratio	52	4–33	High
Phosphorus	6.7 mg/dL	2.4–8.2 mg/dL	
Glucose	124 mg/dL	64–170 mg/dL	
Calcium	7.8 mg/dL	8.2–10.8 mg/dL	Low
Magnesium	2.5 mEq/L	1.5–2.5 mEq/L	
Sodium	144 mEq/L	145–158 mEq/L	Low
Potassium	3.5 mEq/L	3.4–5.6 /µL	
Na/K Ratio	41	32–41	
Chloride	112 mEq/L	104–128 mEq/L	
Cholesterol	62 mg/dL	75–220 mg/dL	Low
Triglyceride	160 mg/dL	25–160 mg/dL	
Amylase	989 IU/L	100–1200 IU/L	
Precision PSL	20 U/L	8–26 U/L	
CPK	204 IU/L	56–529 IU/L	
